# Supplementary material for: Current evidence and future direction on evaluating the anticancer effects of curcumin, gingerols, and shogaols in cervical cancer: A systematic review
Source: PLoS One. 2024 Nov 22;19(11):e0314280. doi: 10.1371/journal.pone.0314280 (PMC11584093; doi:10.1371/journal.pone.0314280)
Supplement: S1 Table — (DOCX) [file pone.0314280.s001.docx]

**S1 Table. Risk of bias assessment of *in-vitro* studies.**

| Author/ Year/ Country | 1. Was the cancer cell lines used reported? | 2. Was the duration of exposure to the cancer cell line reported? | 3. Was the concentration on the cancer cell culture employed reported? | 4. Was a standard culture media used for treatment control? | 5. Were reliable tools used to assess the outcome? | 6. Were the experiments conducted in triplicate? | 7. Were more than one independent experiment performed? |
| --- | --- | --- | --- | --- | --- | --- | --- |
| Chakrabourty et al./ 2012/ India [1] | Yes | Yes | Yes | Yes | Yes | Yes | Yes |
| Chearwae et al./ 2004/ Thailand [2] | Yes | Yes | Yes | Yes | Yes | Yes | Yes |
| Debata et al./ 2013/ USA [3] | Yes | Yes | No | Yes | Yes | Yes | Yes |
| Divya et al./ 2006/ India [4] | Yes | Yes | Yes | Yes | Yes | Yes | Yes |
| Ghasemi et al./ 2019/ Iran [5] | Yes | Yes | Yes | Yes | Yes | Unclear | Yes |
| Kapoor et al./ 2016/ India [6] | Yes | Yes | Yes | Yes | Yes | Yes | Unclear |
| Lewinska et al./ 2014/ Poland [7] | Yes | Yes | Yes | Yes | Yes | Unclear | Yes |
| Limtrakul et al./ 2004/ Thailand [8] | Yes | Yes | No | Yes | Yes | Yes | Yes |
| Liu et al./ 2012/ China [9] | Yes | Yes | Yes | Yes | Yes | Yes | Yes |
| Madden et al./ 2009/ USA [10] | Yes | Yes | Yes | Yes | Yes | Unclear | Yes |
| Maher et al./ 2011/ USA [11] | Yes | Yes | Yes | Yes | Yes | Yes | Yes |
| Mohammad Noor et al./ 2020/ Malaysia [12] | Yes | Yes | Yes | Yes | Yes | Yes | Unclear |
| Patiño-Morales et al./ 2020/ Mexico [13] | Yes | Yes | Yes | Yes | Yes | Unclear | Yes |
| Pei et al./ 2021/ China [14] | Yes | Yes | Yes | Yes | Yes | Unclear | Yes |
| Prusty/ 2005/ India [15] | Yes | Yes | No | Yes | Yes | Unclear | Unclear |
| Raghav/ 2018/ India [16] | Yes | Yes | Yes | Yes | Yes | Unclear | Yes |
| Rastogi/ 2015/ India [17] | Yes | Yes | Yes | Yes | Yes | Unclear | Yes |
| Ruangnoo et al./ 2012/ Thailand [18] | Yes | Yes | Yes | Yes | Yes | Yes | Yes |
| Shang et al./ 2016/ China [19] | Yes | Yes | Yes | Yes | Yes | Unclear | Yes |
| Singh et al./ 2009/ India [20] | Yes | Yes | Yes | Yes | Yes | Unclear | Yes |
| Singh et al./ 2011/ India [21] | Yes | Yes | Yes | Yes | Yes | Unclear | Yes |
| Wang et al./ 2020/ China [22] | Yes | Yes | Yes | Yes | Yes | Yes | Yes |
| Zhang et al./ 2017/ China [23] | Yes | Yes | Yes | Yes | Yes | Yes | Yes |
| Zhang et al./ 2017/ China [24] | Yes | Yes | Yes | Yes | Yes | Yes | Yes |

**References**

1. Chakraborty D, Bishayee K, Ghosh S, Biswas R, Mandal SK, Khuda-Bukhsh AR. [6]-Gingerol induces caspase 3 dependent apoptosis and autophagy in cancer cells: Drug-DNA interaction and expression of certain signal genes in HeLa cells. European Journal of Pharmacology. 2012;694(1-3):20-9.

2. Chearwae W, Anuchapreeda S, Nandigama K, Ambudkar SV, Limtrakul P. Biochemical mechanism of modulation of human P-glycoprotein (ABCB1) by curcumin I, II, and III purified from turmeric powder. Biochemical Pharmacology. 2004;68(10):2043-52.

3. Debata PR, Castellanos MR, Fata JE, Baggett S, Rajupet S, Szerszen A, et al. A novel curcumin-based vaginal cream Vacurin selectively eliminates apposed human cervical cancer cells. Gynecology Oncology. 2013;129(1):145-53.

4. Divya CS, Pillai MR. Antitumor action of curcumin in human papillomavirus associated cells involves downregulation of viral oncogenes, prevention of NFkB and AP-1 translocation, and modulation of apoptosis. Molecular Carcinogenesis. 2006;45(5):320-32.

5. Ghasemi F, Shafiee M, Banikazemi Z, Pourhanifeh MH, Khanbabaei H, Shamshirian A, et al. Curcumin inhibits NF-kB and Wnt/beta-catenin pathways in cervical cancer cells. Pathology- Research Practice. 2019;215(10):152556.

6. Kapoor V, Aggarwal S, Das SN. 6-Gingerol mediates its anti tumor activities in human oral and cervical cancer cell lines through apoptosis and cell cycle arrest. Phytotherapy Researh. 2016;30(4):588-95.

7. Lewinska A, Adamczyk J, Pajak J, Stoklosa S, Kubis B, Pastuszek P, et al. Curcumin-mediated decrease in the expression of nucleolar organizer regions in cervical cancer (HeLa) cells. Mutation Research/Genetic Toxicology and Environmental Mutagenesis. 2014;771:43-52.

8. Limtrakul P, Anuchapreeda S, Buddhasukh D. Modulation of human multidrug-resistance MDR-1 gene by natural curcuminoids BMC Cancer. 2004;4(13).

9. Liu Q, Peng Y, Qi L, Cheng X, Xu X, Liu L, et al. The cytotoxicity mechanism of 6-shogaol-treated HeLa human cervical cancer cells revealed by label-free shotgun proteomics and bioinformatics analysis. Evid -Based Complementary Altern Med. 2012;2012.

10. Madden K, Flowers L, Salani R, Horowitz I, Logan S, Kowalski K, et al. Proteomics-based approach to elucidate the mechanism of antitumor effect of curcumin in cervical cancer. Prostaglandins, Leukotrienes & Essential Fatty Acids. 2009;80(1):9-18.

11. Maher DM, Bell MC, O'Donnell EA, Gupta BK, Jaggi M, Chauhan SC. Curcumin suppresses human papillomavirus oncoproteins, restores p53, Rb, and PTPN13 proteins and inhibits benzo[a]pyrene-induced upregulation of HPV E7. Mol Carcinog. 2011;50(1):47-57.

12. Mohammad Noor HS, Sukari MA, Ismail IS, Abdul AB. In vitro cytotoxic, radical scavenging and antimicrobial activities of curcuma mangga valeton and van zijp. International Journal of Medical Toxicology & Legal Medicine. 2020;23(1 and 2).

13. Patiño-Morales CC, Soto-Reyes E, Arechaga-Ocampo E, Ortiz-Sanchez E, Antonio-Vejar V, Pedraza-Chaverri J, et al. Curcumin stabilizes p53 by interaction with NAD(P)H:quinone oxidoreductase 1 in tumor-derived cell lines. Redox Biology. 2020;28:101320.

14. Pei X, He Z, Yao H, Xiao J, Li L, Gu J, et al. 6-shogaol from ginger shows anti-tumor effect in cervical carcinoma via Pl3K/Akt/mTOR pathway. Eur J Nutr. 2021;60:2781-93.

15. Prusty BK, Das BC. Constitutive activation of transcription factor AP-1 in cervical cancer and suppression of human papillomavirus (HPV) transcription and AP-1 activity in HeLa cells by curcumin. International Journal of Cancer. 2004;113(6):951-60.

16. Raghav D, Sebastian J, Rathinasamy K. Biochemical and biophysical characterization of curcumin binding to human mitotic kinesin Eg5: Insights into the inhibitory mechanism of curcumin on Eg5. International Journal of Biological Macromolecules. 2018;109:1189-208.

17. Rastogi N, Duggal S, Singh S, Porwal K, Srivastava V, Maurya R, et al. Proteasome inhibition mediates p53 reactivation and anti-cancer activity of 6-Gingerol in cervical cancer cells Oncotarget. 2015;6(41):43310-25.

18. Ruangnoo S, Itharat A, Sakpakdeejaroen I, Rattarom R, Tappayutpijarn P, Pawa K. In vitro cytotoxic activity of Benjakul herbal preparation and its active compounds against human lung, cervical and liver cancer cells. Journal of Medical Association of Thailand. 2012;95:127-34.

19. Shang H, Chang C, Chou Y, Yeh M, Au M, Lu H, et al. Curcumin causes DNA damage and affects associated protein expression in HeLa human cervical cancer cells Oncol Rep. 2016;36:2207-15.

20. Singh M, Singh N. Molecular mechanism of curcumin induced cytotoxicity in human cervical carcinoma cells. Molecular and Cellular Biochemistry. 2009;325:107-19.

21. Singh M, Singh N. Curcumin counteracts the proliferative effect of estradiol and induces apoptosis in cervical cancer cells Molecular and Cell Biochemistry 2011;347:1-11.

22. Wang T, Wu X, Al Rudaisat M, Song Y, Cheng H. Curcumin induces G2/M arrest and triggers autophagy, ROS generation and cell senescence in cervical cancer cells. Journal of Cancer. 2020;11(22):6704-15.

23. Zhang F, Thakur K, Hu F, Zhang JG, Wei ZJ. 10-Gingerol, a phytochemical derivative from "Tongling white ginger", inhibits cervical cancer: Insights into the molecular mechanism and inhibitory targets. Journal of Agricultural and Food Chemistry. 2017a;65(10):2089-99.

24. Zhang F, Zhang JG, Qu J, Zhang Q, Prasad C, Wei ZJ. Assessment of anti-cancerous potential of 6-gingerol (Tongling white ginger) and its synergy with drugs on human cervical adenocarcinoma cells. Food and Chemical Toxicology. 2017b;109(Pt 2):910-22.
