# Supplementary material for: Current evidence and future direction on evaluating the anticancer effects of curcumin, gingerols, and shogaols in cervical cancer: A systematic review
Source: PLoS One. 2024 Nov 22;19(11):e0314280. doi: 10.1371/journal.pone.0314280 (PMC11584093; doi:10.1371/journal.pone.0314280)
Supplement: S2 Table — (DOCX) [file pone.0314280.s002.docx]

**S2 Table. Risk of bias assessment of *in-vivo* studies.**

| Author/Year/ Country | 1. Was the allocation sequence adequately generated and applied? | 2. Were the groups similar at baseline or adjusted for confounders in the analysis? | 3. Was the allocation to the different groups adequately concealed? | 4. Were the animals randomly housed during experiments? | 5. Were the caregivers and/or investigator blinded from the knowledge which intervention each animal received during the experiment? | 6. Were the animals selected random for outcome assessment? | 7. Was the outcome assessor blinded? | 8. Were incomplete outcome data adequately addressed? | 9. Are reports of the study free of selective outcome reporting? | 10. Was the study apparently free of other problems that could result in a high risk of bias? |
| --- | --- | --- | --- | --- | --- | --- | --- | --- | --- | --- |
| Pei et al./ 2021/ China [1] | Unclear | Yes | Yes | Unclear | Unclear | Unclear | Unclear | Yes | Yes | Unclear |
| Rastogi et al./ 2015/ India [2] | Unclear | Yes | Yes | Unclear | Unclear | Unclear | Unclear | Yes | Yes | Yes |
| Yoysungnoen-Chintana et al./ 2014/ Thailand [3] | Unclear | Yes | Yes | Unclear | Unclear | Unclear | Unclear | Yes | Yes | Unclear |

**References**

1. Pei X, He Z, Yao H, Xiao J, Li L, Gu J, et al. 6-shogaol from ginger shows anti-tumor effect in cervical carcinoma via Pl3K/Akt/mTOR pathway. Eur J Nutr. 2021;60:2781-93.

2. Rastogi N, Duggal S, Singh S, Porwal K, Srivastava V, Maurya R, et al. Proteasome inhibition mediates p53 reactivation and anti-cancer activity of 6-Gingerol in cervical cancer cells Oncotarget. 2015;6(41):43310-25.

3. Yoysungnoen-Chintana P, Bhattarakosol P, Patumraj S. Antitumor and antiangiogenic activities of curcumin in cervical cancer xenografts in nude mice. Biomed Research International. 2014;2014:817972.
