# Supplementary material for: Current evidence and future direction on evaluating the anticancer effects of curcumin, gingerols, and shogaols in cervical cancer: A systematic review
Source: PLoS One. 2024 Nov 22;19(11):e0314280. doi: 10.1371/journal.pone.0314280 (PMC11584093; doi:10.1371/journal.pone.0314280)
Supplement: S3 Table — (DOCX) [file pone.0314280.s003.docx]

| **S3 Table: Summary of included studies.** | | | | | | | | |
| --- | --- | --- | --- | --- | --- | --- | --- | --- |
| ***In-vitro* studies** | | | | | | | | |
| **Author (Year)/ Country of origin** | | **Type of cell (n=)** | **Intervention of interest** | | **Assay** | **Outcome measurement** | | **Comparator/ control** |
|  |  |  | **Dosage*** | **Duration** |  | **Anti-cancer activities & Cytotoxicity effects** | **Inhibition concentration profile** |  |
| **Curcumin** | | | | | | | | |
| 1 | Chearwae et al. (2004)/ Thailand [1] | KB-V1 and KB-3-1 (n=3) | 0-100 µM | 72 hr | MTT assay, fluorescent drug accumulation assay, ATPase assay, and photoaffinity labelling of Pgp | At 72 hr, Cur reduced the number of cells KB-V1 and KB-3-1 in dose-dependent manner. Cur showed an effective MDR modulator by modulating the function of Pgp, an efflux pump that transports the drug substrate. Cur increased the sensitivity to vinblastine and retained the drug more effectively in carcinoma cells. | IC_50_= 23.5 ± 5.6 µM (KB-V1) and 24.0 ± 1.7 µM (KB-3-1) | DMEM with 10% fetal calf serum |
| 2 | Debata et al. (2013)/ USA [2] | HeLa, ME-180, SiHa, and SW756 (n=2) | 0-100 µM | 96 hr | WST-1 assay, immunofluorescence staining, and WB | At 96 hr, Cur caused suppression of cancer cell growth. Curcumin eliminated a variety of HPV cervical cancer cells and caused a significant decrease in E6 expression. | IC_50_= 17µM (HeLa), 12µM (ME-180), 51µM (SiHa) and 21µM (SW756) | DMEM with 10% FBS |
| 3 | Divya et al. (2006)/ India [3] | Hela, SiHa, and C33A (n=3) | 5-60 µM | 24, 48, and 60 hr | MTT assay, Acridine Orange-Ethidium Bromide Staining method, DNA fragmentation assay, TUNEL assay, RT-PCR, WB, and EMSA | Cur inhibited cancer cell proliferation and showed a gradual increment in sensitivity. Cur induced apoptosis and DNA (nuclear and internucleosomal) fragmentation. Expression of viral oncogenes E6 and E7 was inhibited. | Not mentioned | DMEM with 10% FBS |
| 4 | Ghasemi et al. (2019)/ Iran [4] | HeLa (n=3) | 0.0001-1000 µM/mL | 24 and 48 hr | MTT assay, cell cycle assay, RT-PCR, and invasion assay | Cur inhibited cancer cell growth, enhanced cell death, decreased transwell invasion, and shrinkage of tumour size after 7 days. Cur caused G_2_/M cell cycle arrest followed by sub-G1 apoptosis. | IC_50_= 34.23 µM/mL | 5-Fluorouracil and DMEM with 10% FBS |
| 5 | Lewinska et al. (2014)/ Poland [5] | HeLa (n=5) | 0.01-100 µM | 24 hr | MTT assay, cell cycle analysis, CBMN assay, intracellular superoxide level analysis, AgNOR analysis, DNA methylation analysis, and southern blot | Cur caused changes in cell morphology, decreased in cell metabolic activity and cell viability, and caused cell cycle arrest at G_2_/M. Cell proliferation was decreased with reduction in nuclear division index (NDI) value. Cur as low as 5 µM induced apoptosis by 5-fold and superoxide level (oxidative stress) by 65%. | IC_50_= 8 µM | DMSO |
| 6 | Limtrakul et al. (2004)/ Thailand [6] | KB-V1 and KB-3-1 (n=3) | 1-10 µM | 72 hr | WB and RT-PCR | Cur showed ability as an MDR modulator in multidrug-resistant human cervical cancer cells. | Not mentioned | DMSO |
| 7 | Madden et al. (2009)/ USA [7] | HeLa (n=3) | 0-50 µM | 24, 48 and 72 hr | MTT assay, enzyme immunoassay, 2-dimensional gel electrophoresis, and IPA | Cur inhibited cell proliferation and caused alteration of significant proteins, which were associated with cancer formation and progression. | IC_50_= 15 µM | DMSO |
| 8 | Maher et al. (2011)/ USA [8] | HeLa, SiHa, CaSki, C33A, and SW756 (n=3) | 10-80 µM | 48 hr | Cell proliferation assay, colony forming assay, cell motility assay, 7-AAD assay, WB, and RT-PCR | Cur suppressed cancer cell growth, induced apoptosis, and decreased clonogenic potential and cell motility. Cur inhibited cell growth by reducing cell epithelium thickness. | Not mentioned | DMSO |
| 9 | Martins de Olivera et al. (2023)/ Brazil [9] | HeLa (n=3) | 10-50 µM | 24, 48 and 72 hr | Resazurin assay, cell proliferation recovery assay, spheroid analysis, clonogenic assay, comet assay, and RT-qPCR | Cur inhibited the proliferation of spheroid growth of HeLa cells, decreased cell survival and reduced the formation of new colonies. | IC_50_= 20.61 µM | Doxorubicin and DMSO |
| 10 | Mohammad Noor et al. (2020)/ Malaysia [10] | HeLa | 0.47-30 µg/mL | 72 hr | MTT assay, DPPH radical scavenging assay | Cur showed strong cytotoxicity and antioxidant activity on HeLa cells. | IC_50_= 7.12 ± 0.16 µg/mL | RPMI 1640 with 10% fetal calf serum |
| 11 | Patiño-Morales et al. (2020)/ Mexico [11] | HeLa, SiHa, and CaSki (n=3) | 10-20 µM | 24 hr | MTT assay, WB, and immunoprecipitation assay | Cur promoted stability and restoration of p53 protein via interaction with NQO1 protein, decreasing cell viability of cervical cancer cells. | Not mentioned | DMEM with 10% FBS |
| 12 | Prusty et al. (2005)/ India [12] | HeLa | 50- 200 µM | 24 hr | Northern blot, EMSA assay, and WB | At 2.5 hr, Cur started to selectively suppress HPV 18-specific mRNA expression in Hela cells. | Not mentioned | DMEM with 10% fetal calf serum |
| 13 | Raghav et al. (2018)/ India [13] | HeLa (n=3) | 0-100 µM | 24hr | SRB assay, mitotic index analysis, spectrofluorometric analysis, binding site competition assay, ATPase assay, microtubule sedimentation assay, and synchronous fluorescence spectroscopic | Cur caused mitotic arrest with monopolar spindles, causing inhibition of cell proliferation. Cur binds to Eg5 at a novel site and inhibits the function by structural modifications leading to the monopolar spindle formation in HeLa. | IC_50_= 14 µM | Monastrol and MEM with 10% FBS |
| 14 | Shang et al. (2016)/ China [14] | HeLa (n=3) | 0-14 µM | 48 hr | Cellular viability assay, comet assay, TUNEL assay, and WB | Cur caused morphological changes and decreased HeLa viable cells by inducing comet tail (DNA damage) production, chromatin condensation, and DNA fragmentation in time-dependent manner. | Not mentioned | DMEM with 10% FBS |
| 15 | Singh et al. (2009) /India [15] | HeLa, SiHa, and CaSki (n=3) | 50-100 µM | 24hr | TUNEL assay, telomerase activity assay, WB, and caspase activity assay | Cur induces apoptosis and causes suppression in telomerase activity. | Not mentioned | DMEM and RPMI1640 with 10% FBS |
| 16 | Singh et al. (2011)/ India [16] | HeLa, SiHa, CaSki, and C33A (n=3) | 25-50 µM | 24 hr | Telomerase activity assay, immunohistochemistry, and WB | Cur counteracted the proliferative response of estradiol and induced apoptosis. Cur did not affect telomerase activity in oestradiol primed HPV-positive cancer cells, however significantly decreased it in HPV-negative cancer cells. | Not mentioned | DMEM and RPMI 1640 with 10% FBS |
| 17 | Wang et al. (2020)/ China [17] | SiHa (n=3) | 0-50 µM | 24 hr | CCK-8 assay, monitoring autophagic flux, WB, apoptosis analysis, cell cycle analysis, and SA-beta-gal assay | Cur inhibited cell proliferation and induced ROS accumulation, apoptosis, autophagy, autophagy flux, cell cycle arrest at G_2_/M phase, and cellular senescence in SiHa cells. | Not mentioned | DMEM with 10% FBS |
| 18 | Zhao et al. (2023)/ China [18] | HeLa and CaSki (n=3) | 20-80 µM | 24 hr | MTT assay, Annexin V-FITC/PI, cell migration assay, wound healing assay, and WB | Cur regulated apoptosis, proliferation, and migration of HeLa and CaSki. SiE6 cells (CaSki and HeLa transduced with siE6 lentivirus) treated with Cur showed an offset effect, indicating that the presence of E6 mediates the effects of Cur on cervical cancer cells. | IC_50_= 30.50 µM (HeLa) and 47.8 µM (CaSki) | DMEM with 10% FBS |
| **6-gingerol** | | | | | | | | |
| 19 | Chakrabourty et al. (2012)/ India [19] | HeLa (n=6) | 25 - 175 µg/mL | 24 and 48 hr | MTT assay, DNA fragmentation assay, TUNEL assay, MMP assay, immunoblot, cytochrome c activity assay, Annexin V-FITC/PI, and RT-PCR | 6-G induced cell death by autophagy and caspase 3 mediated apoptosis 6-G reduced HeLa cell viability and changed the cellular and nuclear morphology, including cell shrinkage and membrane blebbing. The chromatin was condensed, leading to HeLa's DNA fragmentation. | IC_50_= 126.89 µg/mL at 24hr and 114.58 µg/mL at 48 hr | DMEM with 10% FBS |
| 20 | Kapoor et al. (2016)/ India [20] | HeLa | 0-800 µM | 48 hr | MTS assay, Annexin V-FITC/PI assay, caspase activity assay, and cell cycle analysis | 6-G caused morphology changes in cancer cells, including cell shrinkage, detachment and membrane blebbing. 6-G affects cell proliferation, induced cell death apoptosis and cell cycle arrest. 6-G caused a robust accumulation of tumour cells in the G_2_ phase from 6.7% to 31.2%. | IC_50_=636 nM | DMEM with 10% fetal calf serum |
| 21 | Ruangnoo et al. (2012)/ Thailand [21] | HeLa (n=3) | 0.1-100 µg/mL | 72 hr | SRB assay | 6-G decreased the HeLa cell viability. | IC_50_= 99.05 µM | MEM with 10% FBS |
| 22 | Zhang et al. (2017)/ China [22] | Hela (n=3) | 25-200 µM | 48 hr | CCK-8 assay, cell cycle analysis, Annexin V-FITC/PI, RT-qPCR, WB | 6-G inhibited cell proliferation, induced apoptosis, and cell cycle arrest at G0/G1- phase. | IC_50_= 96.32 µM | 5-Fluouracil and DMEM with 10% FBS |
| **10-gingerol** | | | | | | | | |
| 23 | Zhang et al. (2017)/ China [23] | HeLa (n=3) | 5-120 µM | 48 hr | CCK-8 assay, cytotoxicity LDH assay, cell cycle analysis, Annexin V-FITC/PI, RT-qPCR, and WB | 10-G altered cancer cell morphology, increased cytotoxicity, and induced cell cycle arrest at the G0/G1 phase. | IC_50_= 29.19 µM | 5-Fluouracil and RPMI 1640 with 10% FBS |
|  | **6-shogaol** | | | | | | | |
| 24 | Liu et al. (2012)/ China [24] | HeLa (n=3) | 5-80 µM | 24 hr | MTT assay, Annexin V-FITC/PI assay, cell cycle analysis, WB, caspase 3 activity assay, JC-1 assay, and IPA | 6-S inhibited HeLa cell proliferation, increased apoptosis rate (early and late apoptosis) up to 53%, and induced cell cycle arrest at the G2 phase. 6-S treated cells displayed a variety of apoptotic cell death responses. | IC_50_= 14.75 ± 0.94 µM | Doxorubicin and DMEM with 10% FBS |
| ***In-vivo* studies** | | | | | | | | |
| **Author (Year)/ Country of origin** | | **Type of cell/ Type of rodent (n=)** | **Intervention of interest** | | **Assay** | **Outcome measurement** | | **Comparator/ control** |
|  |  |  | **Dosage** | **Duration** |  | **Anti-cancer activities & Cytotoxicity effects** | |  |
| **Curcumin** | | | | | | | | |
| 25 | Yoysungnoen-Chintana et al. (2014)/ Thailand [25] | CaSki xenograft male BALB/c-nude mice with weight 20-25g (n=6) | 500-1,500 mg/kg | 30 days | Immunohistochemistry and staining analysis | Cur reduced tumour volume and showed anti-angiogenesis effects. At high doses, Cur reduced the neocapillaries network and induced abnormal patterns. | | MEM |
| ***In-vitro* and *in-vivo* studies** | | | | | | | | |
| **Author (Year)/ Country of origin** | | **Type of rodent (n=)** | **Intervention of interest** | | **Assay** | **Anti-cancer activities & Cytotoxicity effects** | | **Comparator/ control** |
|  |  |  | **Dosage** | **Duration** |  |  |  |  |
| **6-gingerol** | | | | | | | | |
| 26 | Rastogi et al. (2015)/ India [26] | HeLa, CaSki, and SiHa (n=3) | 0-200 µM | 24, 48, and 72 hr | MTT assay, Annexin V-FITC/PI assay, cell cycle analysis, RT-PCR, p53 transactivation assay, WB, and proteasome activity assay | 6-G inhibited cancer cell proliferation through proteasome inhibition mediated p53 reactivation, induced DNA damage and apoptosis, and G_2_/M cell cycle arrest. 6-G potentiated the cytotoxicity of cisplatin in cervical cancer cells. | | DMEM, RPMI 1640 and MEM with 10% FBS |
|  |  | HeLa xenograft male (nu/nu) nude mice at 5–6-week-old (n=6) | 2-5 mg/kg | 45 days | Immunohistochemistry, lipid peroxidation MDA assay | 6-G induced a significant reduction of tumour volume, tumour weight, proteasome inhibition, and p53 accumulation. | | Vehicle (not specified) |
| **6-shogaol** | | | | | | | | |
| 27 | Pei et al. (2021)/ China [27] | HeLa and SiHa (n=3) | 0-80 µM | 12, 24, and 48 hr | CCK-8 assay, colony formation assay, cell cycle analysis, Annexin V-FITC/PE analysis, JC-1 assay, ROS production assay, cell migration assays, and WB | IC_50_= 25.68 ± 0.47 µM (HeLa) and 37.52 ± 1.56 µM (SiHa)  6-S reduced the mitochondrial membrane potential, enhanced apoptosis, and inhibited cell migration and proliferation. 6-S blocked the PI3k/AKT/mTOR pathway and produced cell cycle arrest at the G_2_/M phase. | | DMEM with 10% FBS |
|  |  | HeLa xenograft female BALB/c 18 nude mice at 4-week-old (n=6) | 0-50 mg/kg | 21 days | Immunohistochemistry | 6-S inhibited the tumour growth and induced apoptosis with no obvious side effects (loss of body weight and damage to important organs). | | Normal saline with Tween-80 |

|  |
| --- |

Cur: Curcumin, 6-G: 6-gingerol, 10-G: 10-gingerol, 6-S: 6-shogaol, MTT: 3-(4,5-Dimethylthiazol-2-yl)-2,5-Diphenyltetrazolium Bromide, FACS: Fluorescent drug accumulation assay, WST-1: 4-[3-(4-Iodophenyl)-2-(4-nitrophenyl)-2H-5-tetrazolio]-1,3-benzene Disulfonate, TUNEL: Terminal deoxynucleotide transferase dUTP nick end labelling, RT-PCR: Real time-polymerase chain reaction, EMSA: Electrophoretic mobility shift assay, CBMN: Cytokinesis-block micronucleus, AgNOR: Silver nucleolar organizer regions, IPA: Ingenuity pathway analysis, 7-AAD: 7-amino-actinomycin D, DPPH: 2,2-diphenylpicrylhydrazyl, SRB: Sulforhodamine B, MTS: 3-(4,5-Dimethylthiazol-2-yl)-5-(3-carboxymethoxyphenyl)-2-(4-sulfophenyl)-2H-tetrazolium, CCK-8: Cell counting Kit 8, LDH: Lactate dehydrogenase, JC-1: tetraethylbenzimidazolylcarbocyanine iodide, MDA: malondialdehyde, MDR: multidrug resistance, Pgp: P-glycoprotein 1, ROS: Reactive oxygen species, DMEM: Dulbecco’s modified eagle’s medium, DMSO: Dimethyl sulfoxide, RPMI: Roswell park memorial institute, MEM: Minimum essential medium, WB: western blot, SA-beta-gal: senescence associated-beta-Galactosidase, DMEM: Dulbecco’s Modified Eagle’s medium, FBS: FBS.

**References**

1. Chearwae W, Anuchapreeda S, Nandigama K, Ambudkar SV, Limtrakul P. Biochemical mechanism of modulation of human P-glycoprotein (ABCB1) by curcumin I, II, and III purified from turmeric powder. Biochemical Pharmacology. 2004;68(10):2043-52.

2. Debata PR, Castellanos MR, Fata JE, Baggett S, Rajupet S, Szerszen A, et al. A novel curcumin-based vaginal cream Vacurin selectively eliminates apposed human cervical cancer cells. Gynecology Oncology. 2013;129(1):145-53.

3. Divya CS, Pillai MR. Antitumor action of curcumin in human papillomavirus associated cells involves downregulation of viral oncogenes, prevention of NFkB and AP-1 translocation, and modulation of apoptosis. Molecular Carcinogenesis. 2006;45(5):320-32.

4. Ghasemi M, Turnbull T, Sebastian S, Kempson I. The MTT Assay: Utility, Limitations, Pitfalls, and Interpretation in Bulk and Single-Cell Analysis. Int J Mol Sci. 2021;22(23).

5. Lewinska A, Adamczyk J, Pajak J, Stoklosa S, Kubis B, Pastuszek P, et al. Curcumin-mediated decrease in the expression of nucleolar organizer regions in cervical cancer (HeLa) cells. Mutation Research/Genetic Toxicology and Environmental Mutagenesis. 2014;771:43-52.

6. Limtrakul P, Anuchapreeda S, Buddhasukh D. Modulation of human multidrug-resistance MDR-1 gene by natural curcuminoids BMC Cancer. 2004;4(13).

7. Madden K, Flowers L, Salani R, Horowitz I, Logan S, Kowalski K, et al. Proteomics-based approach to elucidate the mechanism of antitumor effect of curcumin in cervical cancer. Prostaglandins, Leukotrienes & Essential Fatty Acids. 2009;80(1):9-18.

8. Maher DM, Bell MC, O'Donnell EA, Gupta BK, Jaggi M, Chauhan SC. Curcumin suppresses human papillomavirus oncoproteins, restores p53, Rb, and PTPN13 proteins and inhibits benzo[a]pyrene-induced upregulation of HPV E7. Mol Carcinog. 2011;50(1):47-57.

9. Martins de Oliveira L, Alves de Lima LV, Silva MFd, Felicidade I, Lepri SR, Mantovani MS. Disruption of caspase-independent cell proliferation pathway on spheroids (HeLa cells) treated with curcumin. Journal of Toxicology and Environmental Health, Part A. 2023;86(22):859-70.

10. Mohammad Noor HS, Sukari MA, Ismail IS, Abdul AB. In vitro cytotoxic, radical scavenging and antimicrobial activities of curcuma mangga valeton and van zijp. International Journal of Medical Toxicology & Legal Medicine. 2020;23(1 and 2).

11. Patiño-Morales CC, Soto-Reyes E, Arechaga-Ocampo E, Ortiz-Sanchez E, Antonio-Vejar V, Pedraza-Chaverri J, et al. Curcumin stabilizes p53 by interaction with NAD(P)H:quinone oxidoreductase 1 in tumor-derived cell lines. Redox Biology. 2020;28:101320.

12. Prusty BK, Das BC. Constitutive activation of transcription factor AP-1 in cervical cancer and suppression of human papillomavirus (HPV) transcription and AP-1 activity in HeLa cells by curcumin. International Journal of Cancer. 2004;113(6):951-60.

13. Raghav D, Sebastian J, Rathinasamy K. Biochemical and biophysical characterization of curcumin binding to human mitotic kinesin Eg5: Insights into the inhibitory mechanism of curcumin on Eg5. International Journal of Biological Macromolecules. 2018;109:1189-208.

14. Shang H, Chang C, Chou Y, Yeh M, Au M, Lu H, et al. Curcumin causes DNA damage and affects associated protein expression in HeLa human cervical cancer cells Oncol Rep. 2016;36:2207-15.

15. Singh M, Singh N. Molecular mechanism of curcumin induced cytotoxicity in human cervical carcinoma cells. Molecular and Cellular Biochemistry. 2009;325:107-19.

16. Singh M, Singh N. Curcumin counteracts the proliferative effect of estradiol and induces apoptosis in cervical cancer cells Molecular and Cell Biochemistry 2011;347:1-11.

17. Wang T, Wu X, Al Rudaisat M, Song Y, Cheng H. Curcumin induces G2/M arrest and triggers autophagy, ROS generation and cell senescence in cervical cancer cells. Journal of Cancer. 2020;11(22):6704-15.

18. Zhao X, Zhang R, Song Z, Yang K, He H, Jin L, et al. Curcumin suppressed the proliferation and apoptosis of HPV-positive cervical cancer cells by directly targeting the E6 protein. Phytotherapy Research. 2023.

19. Chakraborty D, Bishayee K, Ghosh S, Biswas R, Mandal SK, Khuda-Bukhsh AR. [6]-Gingerol induces caspase 3 dependent apoptosis and autophagy in cancer cells: Drug-DNA interaction and expression of certain signal genes in HeLa cells. European Journal of Pharmacology. 2012;694(1-3):20-9.

20. Kapoor V, Aggarwal S, Das SN. 6-Gingerol mediates its anti tumor activities in human oral and cervical cancer cell lines through apoptosis and cell cycle arrest. Phytotherapy Researh. 2016;30(4):588-95.

21. Ruangnoo S, Itharat A, Sakpakdeejaroen I, Rattarom R, Tappayutpijarn P, Pawa K. In vitro cytotoxic activity of Benjakul herbal preparation and its active compounds against human lung, cervical and liver cancer cells. Journal of Medical Association of Thailand. 2012;95:127-34.

22. Zhang F, Zhang JG, Qu J, Zhang Q, Prasad C, Wei ZJ. Assessment of anti-cancerous potential of 6-gingerol (Tongling white ginger) and its synergy with drugs on human cervical adenocarcinoma cells. Food and Chemical Toxicology. 2017b;109(Pt 2):910-22.

23. Zhang F, Thakur K, Hu F, Zhang JG, Wei ZJ. 10-Gingerol, a phytochemical derivative from "Tongling white ginger", inhibits cervical cancer: Insights into the molecular mechanism and inhibitory targets. Journal of Agricultural and Food Chemistry. 2017a;65(10):2089-99.

24. Liu Q, Peng Y, Qi L, Cheng X, Xu X, Liu L, et al. The cytotoxicity mechanism of 6-shogaol-treated HeLa human cervical cancer cells revealed by label-free shotgun proteomics and bioinformatics analysis. Evid -Based Complementary Altern Med. 2012;2012.

25. Yoysungnoen-Chintana P, Bhattarakosol P, Patumraj S. Antitumor and antiangiogenic activities of curcumin in cervical cancer xenografts in nude mice. Biomed Research International. 2014;2014:817972.

26. Rastogi N, Duggal S, Singh S, Porwal K, Srivastava V, Maurya R, et al. Proteasome inhibition mediates p53 reactivation and anti-cancer activity of 6-Gingerol in cervical cancer cells Oncotarget. 2015;6(41):43310-25.

27. Pei X, He Z, Yao H, Xiao J, Li L, Gu J, et al. 6-shogaol from ginger shows anti-tumor effect in cervical carcinoma via Pl3K/Akt/mTOR pathway. Eur J Nutr. 2021;60:2781-93.
