# Supplementary material for: Current evidence and future direction on evaluating the anticancer effects of curcumin, gingerols, and shogaols in cervical cancer: A systematic review
Source: PLoS One. 2024 Nov 22;19(11):e0314280. doi: 10.1371/journal.pone.0314280 (PMC11584093; doi:10.1371/journal.pone.0314280)
Supplement: Attachment — Submitted filename: Response to Reviewers.docx [file pone.0314280.s006.docx]

**Response to Reviewers’ Comments**

Dear reviewers,

We appreciate the reviewer's attentive reading of the manuscript and your insightful feedback. The comments have assisted us in clarifying and improving the manuscript. Please find the following answer to each comment (reviewers' remarks are in **black**, and our responses are in **blue**).

**Reviewer #1:** This paper aims to conduct a systematic review and meta analysis of the effect of curcumin, gingerol and shogaol on cervical cancer.

The paper presents a lot of information, but it is very difficult to digest. Table 1 has one line per study. This table should be in the appendix. Instead, please summarize across studies as much as possible. Table 2 presents all the results. It is difficult to understand which results have the most support. Please think about how to make this table more useful.

**(Reply)**

Thank you for your careful reading and for highlighting the context. According to the reviewer’s comments, Table 1 has been reassigned to the appendix as S1 Table, Table 2 has been improved to summarize the study results, and the subsequent Table has been renamed accordingly. A new supplementary file, S1 Table, has been created.

**Revised Manuscript:**

1) Page 11, Line 262-263
2) Page 12, Line 273 & 281
3) Page 40, Line 792-795
4) Page 14-24, Table 2

**Changes done:**

1) The risk of bias (RoB) assessment for the *in-vitro* studies included in this review is presented in Fig 2 and S2 Table.
2) The SYRCLE risk of bias for reporting *in-vivo* studies is shown in Fig 3 and S3 Table.
3) **S1 Table.** Summary of included studies.

**S2 Table.** Risk of bias assessment of *in-vitro* studies.

**S3 Table.** Risk of bias assessment of *in-vivo* studies.

**S4 Table.** PRISMA Checklist.

4) Table 2

.

1. The paper is very broad including in vivo and in vitro, human cell lines and animal populations, any preparation of the compounds of interest, and many different kinds of outcomes. Please justify including all these different kinds of studies in the same review.

**(Reply)**

Thank you for highlighting this matter. This systematic review allows for a multifaceted understanding of how these compounds exert their anticancer effects in cervical cancer, particularly in identifying the molecular mechanisms and pathways being targeted by these bioactive compounds. Curcumin known as a potent anti-inflammatory while gingerol known for its antioxidant and immune-boosting properties. By integrating findings from *in-vitro, in-vivo* studies, this review is able to capture comprehensive insights into their chemotherapeutic potential in regardless of preparation of the compounds of interest. As suggested, we have added this information to the manuscript (as shown below).

**Revised Manuscript:**

Page 6, Line 133-140

**Changes done:**

The primary objective is to systematically appraise and synthesise preclinical data, providing a multifaceted understanding of curcumin, gingerol, shogaol, and/or their combination in exerting their anti-cancer effect in cervical cancer, particularly in identifying the molecular mechanism and pathway being targeted. By integrating findings from *in-vitro* and *in-vivo*, this review is able to capture comprehensive insights into their chemotherapeutic potential with a more detailed understanding of the underlying mechanisms and pathways that these compounds of interest affect, regardless of the preparation of the compounds.

2. What is the rationale for excluding studies of human participants?

**(Reply)**

Thank you for highlighting this matter. This SR focused on preclinical evidence to identify molecular targets. Human studies mainly focus on efficacy and adverse reactions. We have added this information to the manuscript (as shown below).

**Revised Manuscript:**

Page 6, Line 140-143

**Changes done:**

The exclusion of clinical studies in evaluating the anti-cancer effects of curcumin, gingerol and shogaol is justified, as overall human studies primarily focus on efficacy and adverse reactions rather than elucidating the specific molecular targets involved.

3. 12 studies were excluded due to the full text not being obtainable. How many and what attempts were made to secure these articles? Please discuss this as a limitation and consider how results could have been impacted by exclusion of such a high proportion of studies.

**(Reply)**

Thank you for highlighting this matter. Efforts have been made to secure the full text are described in the ‘Results’ section. The limitation was discussed as the risk of publication bias in the ‘Study Limitation’ section.

**Revised Manuscript:**

1) Page 11, Line 255-258
2) Page 32, Line 510-513

**Changes done:**

1) Twelve studies were excluded as the full text was unobtainable. Attempts were made to obtain the article, including reaching out to the authors by emailing them. Aside from that, we also requested institutional library services and direct requests to the authors at the Research Gate community.
2) Furthermore, the exclusion of 12 studies due to unavailability may have hindered a comprehensive evaluation of the included studies and skewed the overall assessment of their chemotherapeutic potentials on the anti-cancer effects of the compound of interest.

4.	What is interesting about Table 2, is that there seems to be no replication. Did any studies use similar enough exposures, similar enough outcomes and come up with similar results?

**(Reply)**

Thank you for highlighting this matter. Table 2 has been improved to summarize the results across the studies, particularly on the type of compound, type of cervical cancer cells used/xenografted on mice, and the intervention in terms of concentration/doses and duration.

**Revised Manuscript:**

Page 14-24

**Changes done:**

Table 2: Summary of the mechanism of signalling pathways and molecular targets of included studies. Concentration, dose, and duration of interventions based on the analysis of signalling pathways and molecular targets in cervical cancer cells.

**Table 2:** **Summary of the mechanism of signaling pathways and molecular targets of included studies.** **Concentration, dose, and duration of interventions based on the analysis of signalling pathways and molecular targets in cervical cancer cells.**

| ***In-vitro* studies** | | | | | | | | | | | |
| --- | --- | --- | --- | --- | --- | --- | --- | --- | --- | --- | --- |
| **Compound** | **Cell type** | **Intervention** | | | | | **Mechanism of signalling & molecular targets** | | **References** | |  |
|  |  | **Concentration** | | | **Duration** | |  |  |  |  |  |
| **Curcumin** | **KB-V1** | 0-30 μM | | | 72 hr | | ↓ MDR-I gene expression  ↓ Pgp  ↑ Pgp substrate; calcein-AM, rhodamine123, and bodipy-FL-vinblastine  ↑ ATPase activity | | (1, 2) | |  |
|  | **HeLa** | 0.1-5 μM | | | 24 hr | | ↑ superoxide levels via global DNA hypermethylation  ↑ ROS production by targeting the cytosolic/nuclear thioredoxin system leading to apoptosis | | (3) | |  |
|  |  | 0-15 μM | | | 24-48 hr | | ↓ Ezrin (VIL2) protein  ↓ Phosphoglycerate kinase (PGK1)  ↑ stress-induced phosphoprotein (STIP)  ↑ Rad50 protein  ↑ enolase isoforms 7402 (ENO1 7420 and ENO1 7407)  ↑ UROD and FSIP2 protein expression  DNA damage response | | (4) | |  |
|  |  | 5-20 μM | | | 24 hr | | ↓ E6-associated protein (E6AP)-p53 interaction  ↓ E6 and E7 oncogene expression  ↓ COX-2  ↓ TNF-α induced NF-κB activation  ↑ NQ01  ↑ p21 and p53  Activation of Keap1/Nrf2 pathway  Cytotoxicity via NQO1-p53 complex formation  Prevents AP-1 binding  Inactivation of NF-κB pathway | | (5, 6) | |  |
|  |  | 10-15 μM | | | 18-20 hr | | ↓ ATPase activity of mitotic kinesin Eg5  Inhibits centrosomal separation  Induces mitotic arrest | | (7) | |  |
|  |  | 13 μM | | | 0-48 hr | | ↑ p-ATM and p-ATR  ↑ p53  ↑ MDM2  ↑ BRCA1  ↑ DNA-PK  ↑ MDC1  ↑ p-H2A.X  ↑ PARP  ↑ MGMT protein expression  DNA damage response | | (8) | |  |
|  |  | 20-50 μM | | | 24 hr | | ↓ p16  ↓ oncogene E6 and E7  ↓ PCNA  ↓ cyclin D1  ↓ caspase-3, -9  ↓ Becn1  ↓ PARP1  ↓ Bcl-2  ↓ N-cadherin, E-cadherin, vimentin  ↑ p53, p21 and p73  ↑ Bax | | (9-11) | |  |
|  |  | 34.23 μM | | | 48 hr | | ↓ NF-κB  ↓ Wnt/b-catenin | | (12) | |  |
|  |  | 50 μM | | | 8 hr | | ↓ E6 oncogene  ↓ EGFR  ↓ Rb phosphorylation activation at serine-780 phosphorylation  ↑ p53 | | (13) | |  |
|  |  | 50-100 μM | | | 24 hr | | ↓ ERK and Ras  ↓ cell cycle genes cyclin (D1 and Hsp 70)  ↓ inflammatory proteins (Cox-2 and iNOS)  ↓ p53  ↓ Bcl-2 and Bcl-XL  ↓ C-Myc  ↑ JNK  ↑ Bax  ↑ cytochrome c and caspase-3, -9  Targets MAPK/ERK pathway | | (14) | |  |
|  |  | 100 μM | | | 15 mins- 6 hr | | ↓ p53-responsive gene  ↓ WAF-1/p21 expression  Interfere with AP-1 binding activity via ↓ c-fos and ↑ fra-1 expression | | (15) | |  |
|  | **SiHa** | 5-20 μM | | | 24 hr | | ↓ E6 and E7 oncogene expression  ↓ TNF-α induced NF-κB activation  ↓ E6-associated protein (E6AP)-p53 interaction  ↑ NQ01  ↑ p21 and p53  Activation of Keap1/Nrf2 pathway  Inactivation of NF-κB pathway  Cytotoxicity via NQO1-p53 complex formation | | (5, 6) | |  |
|  |  | 20-50 μM | | | 24-48 hr | | ↓ oncogene E6 and E7 mRNA  ↓ PCNA  ↓ cyclin D1  ↓ G2/M-related genes (cyclins B1 and cdc25)  ↑ cleaved caspase-3 and PARP protein  ↑ p62  ↑ LC3I and LC3II leading to autophagy  ↑ p53 and p21 | | (9, 16, 17) | |  |
|  |  | 50 μM | | | 8 hr | | ↓ oncogene E6  ↓ EGFR  ↑ p53 | | (13) | |  |
|  |  | 50-100 μM | | | 24 hr | | ↓ ERK  ↓ cyclin D1 and Hsp 70  ↓ p53 and p73  ↓ Cox-2 and iNOS  ↓ Bcl-2 and Bcl-XL  ↓ C-Myc  ↑ JNK  ↑ Bax  ↑ cytochrome c and caspase-3, -9  Targets MAPK/ERK pathway | | (14) | |  |
|  | **ME-180** | 50 μM | | | 8 hr | | ↓ E6 oncogene  ↓ EGFR  ↓ Rb phosphorylation activation at serine-780 phosphorylation  ↑ p53 | | (13) | |  |
|  | **SW756** | 50 μM | | | 8 hr | | ↓ E6 oncogene  ↓ EGFR  ↑ p53 | | (13) | |  |
|  | **C33A** | 5-50 μM | | | 24 hr | | ↓ E6 and E7 oncogene expression  ↓ TNF-α induced NF-κB activation  ↓ p16  ↓ cyclin D1  ↑ p53 and p73 gene expression  Inactivation of NF-κB pathway | | (6, 9) | |  |
|  | **CaSki** | 10-50 μM | | | 24 hr | | ↓ E6-associated protein (E6AP)-p53 interaction  ↓ PCNA  ↓ cyclin D1  ↓ oncogene E6 and E7  ↑ NQ01  ↑ p21 and p53  Cytotoxicity via NQO1-p53 complex formation  Activation of Keap1/Nrf2 pathway | | (5, 9) | |  |
|  |  | 20-40 μM | | | 24-48 hr | | ↓ oncogene E6 and E7  ↓ BaP-induced HPV E7  ↑ caspase-3, -9 and cleaved PARP  ↑ p53, pRb, and PTPN13 expression | | (16) | |  |
|  |  | 50-100 μM | | | 24 hr | | ↓ ERK  ↓ cyclin D1 and Hsp 70  ↓ p53 and p73  ↓ C-Myc  ↑ JNK  ↑ Bax, cytochrome c and caspase-3, -9  Targets MAPK/ERK pathway | | (14) | |  |
| **6-gingerol** | **HeLa** | 50 μM | | | 24 hr | | ↓ cyclin B1  ↓ of PARP  ↑ p53 and p21  ↑ p-H2AX  ↑ cleaved caspase-3  Genotoxic stress via MRC-1 inhibition  DNA damage response | | (18) | |  |
|  |  | 60-140 μM | | | 48 hr | | ↓ cell cycle genes (cyclin A, cyclin D1, cyclin E1) and CDK-1  ↓ p21 and p27  ↓ PI3K/AKT pathway  ↑ cytochrome c, Bax, caspase-3, -8, -9, and PARP  ↑ of AMPK | | (19) | |  |
|  |  | 75-125 μg/mL | | | 48 hr | | ↓ NF-κB  ↓ AKT  ↑ caspase 3, PARP, Bax and cytochrome c  ↑ Bcl-2  ↑ TNFα  Induce autophagy death via ↑ acidic vacuoles (AVO) formation | | (20) | |  |
|  | **CaSki** | 50 μM | | | 24 hr | | ↓ cyclin B1  ↓ PARP expressions  ↑ p53 and p21  ↑ p-H2AX  ↑ cleaved caspase-3  Genotoxic stress via MRC-1 inhibition  Oncogene E6 and E7 shows no changes | | (18) | |  |
| **10-gingerol** | **HeLa** | 15-50 μM | | | 48 hr | | ↓ cyclin A, cyclin D1, cyclin E1, CDK-1, CDK-2, CDK-4, CDK-6  ↓ p15, p21, p16, and p27  ↓ GSK-3B  ↓ β-catenin  ↓ Bcl-2  ↓ PI3K/AKT pathway  ↓ mTOR phosphorylation  ↓ NF-κB pathway  ↑ death receptors proteins DR3 and DR5  ↑ cleaved caspase-3, -8, -9, cytochrome c, Bid, Bad, and Bax  ↑ AMPK pathway | | (21) | |  |
| **6-shogaol** | **HeLa** | 20-40 μM | | | 0-24 hr | | ↓ CDC25A  ↓ cyclin B1  ↓ PCNA  ↓ Bcl-2  ↓ p62  ↓ cell migration regulators genes (N-cadherin, MMP-2, and MMP9)  ↓ Snail, Twist, Zeb-1, and Zeb-2 gene expression  ↓ PI3K/AKT/mTOR pathway via ↓ p-PI3K, p-AKT, and p-mTOR  ↓ ER-stress-associated protein (PERK and ARF5)  ↑ cytochrome c, PARP, Bax, and caspase 3  ↑ autophagy-related proteins (LC3-II and Beclin 1)  ↑ E-cadherin  ↑ HSP60  ↑ Annexin A1, cofilin, and calreticulin  No changes in CHOP expression | | (22, 23) | |  |
|  | **SiHa** | 20-40 μM | | | 24 hr | | ↓ Bcl-2  ↓ p62  ↓ cell migration regulators genes (N-cadherin, MMP-2, and MMP9)  ↓ Snail, Twist, Zeb-1, and Zeb-2 gene expression  ↓ PI3K/AKT/mTOR pathway via ↓ p-PI3K, p-AKT, and p-mTOR  ↑ cytochrome c, PARP, and Bax  ↑ autophagy-related proteins (LC3-II and Beclin 1)  ↑ E-cadherin | | (22) | |  |
| ***In-vivo* studies** | | | | | | | | | | | |
| **Compound** | **Cell type** | | **Intervention** | | | **Mechanism of signalling & molecular targets** | | **References** | |  |  |
|  |  |  | **Dosage (mg/Kg)** | **Duration**  **(days)** | |  |  |  |  |  |  |
| **Curcumin** | **CaSki implanted nude mice** | | 1,500 mg/Kg | 0-28 days | | ↓ angiogenesis biomarkers (VEGF, COX-2, EGFR) | | (24) | |  |  |
| **6-gingerol** | **HeLa implanted mice** | | 2.5-5 mg/Kg | 0-30 days | | ↓ Bax, GADD45, Noxa, Puma  ↓ p21  ↑ oxidative stress biomarker (MDA)  ↑ p53 | | (18) | |  |  |

↑: upregulation, ↓: downregulation, MDR-1: multidrug resistance 1, Pgp: phosphoglycolate phosphatase, ATPase: adenosine triphosphatase, ROS: reactive oxygen species, STIP: stress-induced phosphoprotein, PGK1: phosphoglycerate kinase-1, ENO1: enolase 1, UROD: uroporphyrinogen decarboxylase, FSIP2: fibrous sheath interacting protein, Keap1: Kelch-like ECH-associated protein 1, Nrf2: nuclear factor erythroid 2-related factor 2, NQ01: NAD(P)H quinone dehydrogenase 1, E6AP: E6-associated protein, COX-2: cyclooxygenase-2, AP-1: activator protein-1, TNF-α: tumour necrosis factor alpha, NF-κB: nuclear factor kappa B, ATM: ataxia telangiectasia mutated, ATR: ataxia telangiectasia and Rad3-related protein, MDM2: murine double minute 2, BRCA1: breast cancer gene 1, DNA-PK: DNA-dependent protein kinase, MDC1: mediator of DNA damage checkpoint protein 1, p-H2A.X: phosphorylated histone H2A Variant X, PARP: poly(ADP-ribose) polymerase, MGMT: O6-methylguanine-DNA methyltransferase, PCNA: proliferating cell nuclear antigen, Becn1: Beclin 1, Bcl-2: B-cell lymphoma 2, Bax, Bcl-2 associated X protein, N-cadherin: Neural cadherin, E-cadherin; Epithelial cadherin, Wnt: wingless and Integrated-1, EGFR: epidermal growth factor receptor, Rb: retinoblastoma, MAPK: Mitogen-activated protein Kinase, ERK: Extracellular signal-regulated kinase, Ras: Rat sarcoma viral oncogene, JNK: c-Jun N-terminal kinase, Hsp70: Heat shock protein 70, iNOS: inducible nitric oxide synthase, Bcl-xl: B-cell lymphoma extra-large, C-myc: myelocytomatosis oncogene, c-fos: cellular proto-oncogene fos, fra-1: Fos-related antigen 1, WAF-1: wild-type p53-activated fragment, MRC-1: macrophage mannose receptor-1, CDK-1: cyclin-dependent kinase-1, AMPK: adenosine monophosphate-activated protein, AKT: protein kinase B, GSK-3B: glycogen synthase-3 beta, PI3K: phosphoinositide 3-kinase, mTOR: mechanistic target of rapamycin, CDC25A: cell division cycle 25A, MMP: matrix metalloproteinase, Zeb: Zinc finger E-box-binding homeobox, HSP60: heat shock protein 60, CHOP: C/EBP homologous protein, VEGF: Vascular endothelial growth factor, GADD45: growth arrest and DNA damage-inducible 45, MDA: malondialdehyde.

5.	Some statements are made, without evidence presented. "... the highest concentration of these compounds is associated with the lowest cell proliferation in all tested cell lines" Where are these results presented?

**(Reply)**

Thank you for highlighting this matter. As suggested, the sentence has been rephrased and reference to the studies referring to the statements were added as follows:

3. Lewinska A, Adamczyk J, Pajak J, Stoklosa S, Kubis B, Pastuszek P, et al. Curcumin-mediated decrease in the expression of nucleolar organizer regions in cervical cancer (HeLa) cells. Mutation Research/Genetic Toxicology and Environmental Mutagenesis. 2014;771:43-52.

6. Divya CS, Pillai MR. Antitumor action of curcumin in human papillomavirus associated cells involves downregulation of viral oncogenes, prevention of NFkB and AP-1 translocation, and modulation of apoptosis. Molecular Carcinogenesis. 2006;45(5):320-32.

7. Raghav D, Sebastian J, Rathinasamy K. Biochemical and biophysical characterization of curcumin binding to human mitotic kinesin Eg5: Insights into the inhibitory mechanism of curcumin on Eg5. International Journal of Biological Macromolecules. 2018;109:1189-208.

10. Martins de Oliveira L, Alves de Lima LV, Silva MFd, Felicidade I, Lepri SR, Mantovani MS. Disruption of caspase-independent cell proliferation pathway on spheroids (HeLa cells) treated with curcumin. Journal of Toxicology and Environmental Health, Part A. 2023;86(22):859-70.

16. Maher DM, Bell MC, O'Donnell EA, Gupta BK, Jaggi M, Chauhan SC. Curcumin suppresses human papillomavirus oncoproteins, restores p53, Rb, and PTPN13 proteins and inhibits benzo[a]pyrene-induced upregulation of HPV E7. Molecular Carcinogenesis. 2011;50(1):47-57.

18. Rastogi N, Duggal S, Singh S, Porwal K, Srivastava V, Maurya R, et al. Proteasome inhibition mediates p53 reactivation and anti-cancer activity of 6-Gingerol in cervical cancer cells Oncotarget. 2015;6(41):43310-25.

20. Chakraborty D, Bishayee K, Ghosh S, Biswas R, Mandal SK, Khuda-Bukhsh AR. [6]-Gingerol induces caspase 3 dependent apoptosis and autophagy in cancer cells: Drug-DNA interaction and expression of certain signal genes in HeLa cells. European Journal of Pharmacology. 2012;694(1-3):20-9.

22. Pei X, He Z, Yao H, Xiao J, Li L, Gu J, et al. 6-Shogaol from ginger shows anti-tumor effect in cervical carcinoma via Pl3K/Akt/mTOR pathway. European Journal of Nutrition. 2021;60:2781-93.

23. Liu Q, Peng Y, Qi L, Cheng X, Xu X, Liu L, et al. The cytotoxicity mechanism of 6-shogaol-treated HeLa human cervical cancer cells revealed by label-free shotgun proteomics and bioinformatics analysis. Evidance-Based Complementary and Alternative Medicine. 2012;2012.

25. Kapoor V, Aggarwal S, Das SN. 6-Gingerol mediates its anti tumor activities in human oral and cervical cancer cell lines through apoptosis and cell cycle arrest. Phytotherapy Researh. 2016;30(4):588-95.

**Revised Manuscript:**

Page 25

Line 345-347

**Changes done:**

Many studies have reported that the highest concentration of these compounds is associated with the lowest cell proliferation in all tested cell lines (3, 6, 7, 10, 16, 18, 20, 22, 23, 25).

6.	Generally, more care should be taken to present results in a way that highlights where there has been replication.

**(Reply)**

Thank you for highlighting this matter. Table 2 has been revised to emphasized the replication of findings across the studies.

7.	Among the excluded studies are 116 which were review articles. How has this paper improved upon previous attempts at reviewing this literature?

**(Reply)**

Thank you for highlighting this matter. The excluded reviews of similar topics either focused on assessing ginger and turmeric extract, bioactive compounds used alone or their role in other types of cancer or other health condition(s). And many of them are not systematic reviews.

The current systematic review has improved by only focusing on including only studies using bioactive compounds, thus ensuring uniformity of intervention. Plant extract may contain other bioactive compounds; therefore, it is difficult to determine the true anticancer effect. Our search is more recent and updated. Also, we focused on the molecular mechanism targeted by these bioactive compounds, particularly in cervical cancers.

**Revised Manuscript:**

Page 25

Line 331-335

**Changes done:**

The current systematic review has improved upon previous reviews as it refined its focus by including only studies using bioactive compounds, thus ensuring uniformity of intervention. Previous reviews concentrated on specific extracts like ginger and turmeric or for addressing bioactive compounds in different cancer types or other health condition(s), many of which lacked systematic methodologies.

The information is not meta analyzed, but this is the correct decision as there is a lot of heterogeneity in the studies considered

**Reviewer #2:**

The current study The paper evaluates the anticancer effects of curcumin, gingerols, and shogaols specifically in cervical cancer and concludes that these compunds have promising anticancer properties. It can be said that the purpose of this study and its content are attractive and interesting. It offers a good perspective for improving the treatment of cervical cancer with natural products. The article is well written and the design of the study and analyzes are done appropriately and correctly. However, to improve the quality of this study, some major points should be addressed and corrected, which are as follows:

**Major points:**

1. The discussion mentions that most studies included were in vitro, which might not fully represent in vivo conditions. Could you elaborate on how this limitation impacts the generalizability of the findings to clinical settings?

**(Reply)**

Thank you for highlighting this matter. Limitations in *in-vivo* studies may impact the generalizability of findings to clinical settings due to more complex biological systems present in *in-vivo* when compared to *in vitro.* An elaboration of this limitation is added to the ‘Study Limitation’ section (as below).

**Revised Manuscript:**

Page 32, Line 499-510

**Changes done:**

Nevertheless, this systematic review is not without limitations. Most of the included studies employed an *in-vitro* model. *In-vitro* cell culture may not account for the interactions between physiological processes, metabolic processes, and other cellular mechanisms that occur in an organ system when subjected to the studied bioactive compounds. Consequently, the limitations of *in-vitro* studies may impact the generalizability of findings to clinical settings. The limited robustness of preclinical research has been identified as one of the drivers of clinical trial failure (26). Without validation through *in-vivo* studies, findings from *in-vitro* may lead to overestimations or misinterpretations of the efficacy and safety of the treatments when applied to human subjects. Although the overall risk of bias for most in-vitro studies assessed was judged as low, indicating reliable study outcomes, additional *in-vivo* studies are necessary to ascertain the effectiveness and safety profile of the bioactive compounds.

2. The methodological quality of the in vitro studies was assessed as low. How does this low methodological quality affect the reliability of the overall conclusions drawn from the review?

**(Reply)**

Thank you for highlighting this matter. Determining methodological quality for bias in a study conducted is important in determining the validity of research findings. Low bias indicates low concerns about any potential biases and limitations in the research design, execution, and reporting. It highlights transparency and clarity in the studies. Consequently, it strengthens the conclusions drawn from the review. The sentences have been rephrased accordingly.

**Revised Manuscript:**

Page 32, Line 508-510

**Changes done:**

Although the overall risk of bias for most *in-vitro* studies assessed were judged as low, indicating reliable study outcomes, additional *in-vivo* studies are necessary to ascertain the effectiveness and safety profile of the bioactive compounds.

3. There any significant inconsistencies in study designs, such as variations in cell lines, exposure times, or concentrations of bioactive compounds, that could have influenced the results. How were these addressed in the review?

**(Reply)**

Thank you for highlighting this matter. Table 1 has been revised to summarize the results across the studies into same group of cell lines, exposure times, and concentrations with same compound treatments.

**Revised Manuscript:**

Page 14-24

**Changes done:**

Table 1

4. Given the challenges in determining an optimal IC50 due to varying cell lines, exposure durations, and assay conditions, how should future research standardize these parameters to enhance comparability?

**(Reply)**

Thank you for highlighting this matter. Recommendation for future studies regarding reporting and parameters of IC_50_ was added to the ‘Future Research Direction’ section.

**Revised Manuscript:**

Page 32-33, Line 518-530

**Changes done:**

In this review, the reporting of IC_50_ values reveal significant variability, indicating inconsistencies of IC_50_, which could result in irreproducible experimental outcomes. The systematic IC_50_ errors caused by uneven cell proliferation were found to be the most significant in terms of their misleading effects (27). It was reported that the IC_50_ values for cisplatin could vary significantly based on cell density and proliferation potential, highlighting the importance of controlling this variable. It was proposed by He and his team that inconsistencies in IC_50_ values are natural properties of cancer cells rather than remedial artifacts (27). Nevertheless, there should be a concerted effort to improve the reporting of IC_50_, especially in pharmacotherapeutic studies, to ensure consistency and reliability in research findings. Previously, Haibe-Kains et al. (28) suggested the establishment of an international standard for definite IC50 in each cancer cell line, whereas, Punyamurtala et al. (29) described density-dependent IC50 variations as a hallmark of cancer cells and proposed that IC50-seeding density slope (ISDS) serve as a standardized method for assessing therapeutic treatment.

5. The study may not have fully explored the potential synergistic effects of combining these compounds with each other or with other therapeutic agents.

**(Reply)**

Thank you for highlighting this matter. Based on the comment, we do agree that this study has not yet fully explored the potential synergetic effects of the combined compound. We have not yet found any publication done on combination with each compound instead of combination with chemotherapy drugs. We have included it in the ‘Result’ section on page 13, line 302-303, the ‘Study Limitations’ section on page 32, line 513-516, and ‘Future Study Direction’ section on page 34, line 547-550.

1) No studies reported the use of bioactive compounds, as stated above, in combination as a treatment intervention.
2) Another limitation is a lack of research on the combination of curcumin, gingerol and/or shogaol in the treatment of cervical cancer. Combination treatment has been shown to enhance cytotoxicity towards cervical cancer cells and reduce the required concentrations of compounds/drugs, leading to fewer side effects.
3) Moreover, the lack of studies investigating the synergistic effects of curcumin, gingerols, and shogaols when used in combination justifies further research. Given their diverse mechanisms of action, it would be imperative to investigate any potential of synergy in the combination treatments and explore their potential for use in other cancer types.

6. Challenges may exist related to the isolation and purification of these compounds for study purposes. How might these challenges not affect the reproducibility and scalability of research findings?

**(Reply)**

Thank you for highlighting this matter. Discussion on the challenges that may exist related to isolation and purification of the compound of interest has been added at ‘Future Research Directions’ section.

**Revised Manuscript:**

Page 33, Line 531-540

**Changes done:**

Challenges may exist related to the isolation and purification of these bioactive compounds due to the complexity of the extraction process involved. Among the 27 studies reviewed, 17 reported using commercially available bioactive compounds (purity ≥95%). Meanwhile, the remaining studies reported self-isolation and purification, with 4 out of 10 studies reporting a purity of ≥90%, indicating a high level of consistency in the quality of compounds used. The focus on bioactive compounds allows for a more consistent evaluation of efficacy across studies if the purity levels are maintained. Therefore, while challenges related to the isolation and purification of these compounds may exist, the focus on the bioactive compounds rather than varying compositions of the extraction yield could minimize the challenges related to the reproducibility and scalability of research findings

7. Were there any potential biases in the selection or interpretation of studies that the authors may not have fully addressed? How might these biases impact the conclusions drawn in the review?

**(Reply)**

Thank you for highlighting this matter. The protocol for the review was registered and published transparently and was followed throughout the review process, with more than two reviewers involved. However, this review may be subject to publication bias due to a high number of exclusion studies resulting from unobtainable full texts, which could potentially skew the overall assessment of the anti-cancer effects of the compounds. Additionally, the review is also subjected to selection bias due to the publication language restriction. This limitation is discussed in the ‘Study Limitation’ section.

**Revised Manuscript:**

1) Page 32, Line 510-512

**Changes done:**

Furthermore, the exclusion of 12 studies due to unavailability may have hindered a comprehensive evaluation of the included studies and skewed the overall assessment of their chemotherapeutic potentials on the anti-cancer effects of the compound of interest.

8. Given the potential for chemoresistant cervical cancer cells to develop, how might one be sure about these bioactive compounds' effectiveness in overcoming resistance mechanisms?

**(Reply)**

Thank you for highlighting this matter. According to studies conducted by Chearwae et al. (2) and Limtrakul et al. (1) curcumin functions as a modulator of multidrug resistance (MDR) by inhibiting the activity of P-glycoprotein (Pgp), which is involved in transporting anti-cancer drug substrate in drug-resistant cell lines, KB-V1. Identifying and understanding the molecular mechanism and the targeted protein involved in chemoresistant is crucial. By targeting specific pathways, such as the dual inhibition of Mcl-1 and Bcl-2, has shown promise in enhancing chemosensitivity in cervical cancer (29). Therefore, it is essential to explore other proteins and pathways that may contribute to overcoming the resistance mechanisms. This could help with the development of targeted therapies that could enhance the efficacy of bioactive compounds against chemoresistant cervical cancer cells. Besides, the recommendation was added to this review as a future research direction to investigate the potential of gingerol and shogaol in modulating MDR in cervical cancer cell lines. Consequently, improves the effectiveness of the bioactive compounds in drug-resistance cancers.

**Revised Manuscript:**

1) Page 34, Line 552-559

**Changes done:**

Identifying and understanding the molecular mechanism and the targeted protein involved in chemoresistant is crucial for developing effective therapeutic strategies. By targeting specific pathways has shown potential to enhance chemosensitivity in cervical cancer. Therefore, it is essential to explore other proteins and pathways that may contribute to overcome the resistance mechanisms. This could help with the development of targeted therapies aimed at improving the efficacy of bioactive compounds against chemoresistant cervical cancer cells. Furthermore, future research should be directed to investigate the potential of gingerol and shogaol in modulating MDR cancer cell lines.

9. As mentioned in this article, curcumin is a natural nutrient with high potential, which is used as a strong anti-inflammatory substance. Please mention the wide use of curcumin in various diseases (infections, agaeing, cancers, and autoimmunities) in the introduction of a discussion and please consider including the following references in this article (doi:10.1007/s11357-024-01092-5, doi: 10.1016/j.lfs.2021.119437, and doi: 10.1016/j.ejphar.2022.175267).

**(Reply)**

Thank you for highlighting this matter. The references have been added according to the suggestion;

30. Tahmasebi S, Saeed BQ, Temirgalieva E, Yumashev AV, El-Esawi MA, Navashenaq JG, et al. Nanocurcumin improves Treg cell responses in patients with mild and severe SARS-CoV2. Life Sci. 2021;276:119437.

31. Izadi M, Sadri N, Abdi A, Zadeh MMR, Jalaei D, Ghazimoradi MM, et al. Longevity and anti-aging effects of curcumin supplementation. Geroscience. 2024;46(3):2933-50.

**Revised Manuscript:**

1) Page 4-5, Line 100-106

**Changes done:**

Curcumin is recognised for its therapeutic effects on several diseases, such as cancer, autoimmune disease, and various inflammatory conditions (30). This is primarily due to its ability to modulate immune responses, making it beneficial in inducing inflammation and regulating immune functions. Recently, it has been shown to exhibit anti-ageing properties by influencing molecular pathways to ageing, such as AMPK, and the inhibition of NF-κB and mTOR, which contributes to delaying age-associated disorders (31).

10. The use of emerging and powerful treatments based on immunotherapy, such as tuberculosis therapy, has also improved the survival of patients. In the introduction section, explain about this type of treatment in a few sentences and consider including the following references (10.1186/s12935-022-02778-6, doi: 10.1007/s12094-020-02490-9, and doi: 10.1186/s12935-023-02923-9).

**(Reply)**

Thank you for highlighting this matter. The references have been added according to the suggestion:

32.	Al-Haideri M, Tondok SB, Safa SH, maleki AH, Rostami S, Jalil AT, et al. CAR-T cell combination therapy: the next revolution in cancer treatment. Cancer Cell International. 2022;22(1):365.

33.	Tahmasebi S, Elahi R, Khosh E, Esmaeilzadeh A. Programmable and multi-targeted CARs: a new breakthrough in cancer CAR-T cell therapy. Clinical and Translational Oncology. 2021;23(6):1003-19

**Revised Manuscript:**

1) Page 4, Line 83-87

**Changes done:**

Recent advancements in CART-T cell therapy in cancer treatment have demonstrated efficacy in various cancers where the immune cells are genetically designed to better recognise and enhance the immune response to eliminate the cancer cells (32). However, its effectiveness in solid tumours remains limited due to the immunosuppressive tumour microenvironment, which represents significant challenges (33).

11. Another important issue that must be addressed is the presence of inflammatory cytokines in the tumor microenvironment and their confrontation with the PPAR marker due to the existence of common signaling pathways (“Activation of PPARγ negatively influences the production of inflammatory cytokines such as tumor necrosis factor-alpha (TNFα), Interleukin (IL)-6, and IL-1β by macrophages”.). Regarding the contrast with this, explain and consider including the following references in “Cancer Cell Proliferation and Viability” or “Cancer Cell Apoptosis” sections (doi: 10.1007/978-3-030-80962-1_25-1 and doi: 10.1155/2008/961753).

**(Reply)**

Thank you for highlighting this matter. After thorough reading, we value the suggestion on references highlighting the role of PPARγ and its modulation on inflammatory cytokines such as TNFα, IL-6 and IL-1β, which indirectly connect to cancer development. However, it seems that this may fall outside the scope of our systematic review. This systematic review aims to make a significant contribution to the field by focusing on collating and critically assessing *in-vitro* and *in-vivo* studies evaluating the anti-cancer effects of curcumin, gingerol, shogaol, and/or their combination specifically for cervical cancer treatments. The primary objective is to systematically appraise and synthesise preclinical data, providing a multifaceted understanding into identifying the molecular mechanism and pathway being targeted by these bioactive compounds as part of their anti-cancer effects. While inflammatory cytokines are relevent in cancer progression, our review focuses more on direct molecular mechanisms by which curcumin, gingerol, and shogaol inhibit the cancer cell proliferation, induce apoptosis, anti-angiogenesis, anti-metastasis, or inhibit cell cycle, as well as pathways involved that are commonly reported in studies, rather than the general inflammatory response. Our review also did not find any studies reporting on the modulation of the PPARγ on the inflammatory cytokines from the bioactive compound treatments.

12. The quality and resolution of all figures are very less, pleases increase their quality.

**(Reply)**

Thank you for highlighting this matter. Figures with improved resolution have been uploaded.

13. There are some grammatical errors that should be corrected throughout the manuscript.

**(Reply)**

Thank you for highlighting this matter. Grammatical errors have been improved.

**References**

1.	Limtrakul P, Anuchapreeda S, Buddhasukh D. Modulation of human multidrug-resistance MDR-1 gene by natural curcuminoids BMC Cancer. 2004;4(13).

2.	Chearwae W, Anuchapreeda S, Nandigama K, Ambudkar SV, Limtrakul P. Biochemical mechanism of modulation of human P-glycoprotein (ABCB1) by curcumin I, II, and III purified from turmeric powder. Biochemical Pharmacology. 2004;68(10):2043-52.

3.	Lewinska A, Adamczyk J, Pajak J, Stoklosa S, Kubis B, Pastuszek P, et al. Curcumin-mediated decrease in the expression of nucleolar organizer regions in cervical cancer (HeLa) cells. Mutation Research/Genetic Toxicology and Environmental Mutagenesis. 2014;771:43-52.

4.	Madden K, Flowers L, Salani R, Horowitz I, Logan S, Kowalski K, et al. Proteomics-based approach to elucidate the mechanism of antitumor effect of curcumin in cervical cancer. Prostaglandins, Leukotrienes & Essential Fatty Acids. 2009;80(1):9-18.

5.	Patiño-Morales CC, Soto-Reyes E, Arechaga-Ocampo E, Ortiz-Sanchez E, Antonio-Vejar V, Pedraza-Chaverri J, et al. Curcumin stabilizes p53 by interaction with NAD(P)H:quinone oxidoreductase 1 in tumor-derived cell lines. Redox Biology. 2020;28:101320.

6.	Divya CS, Pillai MR. Antitumor action of curcumin in human papillomavirus associated cells involves downregulation of viral oncogenes, prevention of NFkB and AP-1 translocation, and modulation of apoptosis. Molecular Carcinogenesis. 2006;45(5):320-32.

7.	Raghav D, Sebastian J, Rathinasamy K. Biochemical and biophysical characterization of curcumin binding to human mitotic kinesin Eg5: Insights into the inhibitory mechanism of curcumin on Eg5. International Journal of Biological Macromolecules. 2018;109:1189-208.

8.	Shang H, Chang C, Chou Y, Yeh M, Au M, Lu H, et al. Curcumin causes DNA damage and affects associated protein expression in HeLa human cervical cancer cells Oncology Reports. 2016;36:2207-15.

9.	Singh M, Singh N. Curcumin counteracts the proliferative effect of estradiol and induces apoptosis in cervical cancer cells Molecular and Cell Biochemistry 2011;347:1-11.

10.	Martins de Oliveira L, Alves de Lima LV, Silva MFd, Felicidade I, Lepri SR, Mantovani MS. Disruption of caspase-independent cell proliferation pathway on spheroids (HeLa cells) treated with curcumin. Journal of Toxicology and Environmental Health, Part A. 2023;86(22):859-70.

11.	Zhao X, Zhang R, Song Z, Yang K, He H, Jin L, et al. Curcumin suppressed the proliferation and apoptosis of HPV-positive cervical cancer cells by directly targeting the E6 protein. Phytotherapy Research. 2023.

12.	Ghasemi F, Shafiee M, Banikazemi Z, Pourhanifeh MH, Khanbabaei H, Shamshirian A, et al. Curcumin inhibits NF-kB and Wnt/beta-catenin pathways in cervical cancer cells. Pathology- Research Practice. 2019;215(10):152556.

13.	Debata PR, Castellanos MR, Fata JE, Baggett S, Rajupet S, Szerszen A, et al. A novel curcumin-based vaginal cream Vacurin selectively eliminates apposed human cervical cancer cells. Gynecol Oncol. 2013;129(1):145-53.

14.	Singh M, Singh N. Molecular mechanism of curcumin induced cytotoxicity in human cervical carcinoma cells. Molecular and Cellular Biochemistry. 2009;325:107-19.

15.	Prusty BK, Das BC. Constitutive activation of transcription factor AP-1 in cervical cancer and suppression of human papillomavirus (HPV) transcription and AP-1 activity in HeLa cells by curcumin. International Journal of Cancer. 2004;113(6):951-60.

16.	Maher DM, Bell MC, O'Donnell EA, Gupta BK, Jaggi M, Chauhan SC. Curcumin suppresses human papillomavirus oncoproteins, restores p53, Rb, and PTPN13 proteins and inhibits benzo[a]pyrene-induced upregulation of HPV E7. Molecular Carcinogenesis. 2011;50(1):47-57.

17.	Wang T, Wu X, Al Rudaisat M, Song Y, Cheng H. Curcumin induces G2/M arrest and triggers autophagy, ROS generation and cell senescence in cervical cancer cells. Journal of Cancer. 2020;11(22):6704-15.

18.	Rastogi N, Duggal S, Singh S, Porwal K, Srivastava V, Maurya R, et al. Proteasome inhibition mediates p53 reactivation and anti-cancer activity of 6-Gingerol in cervical cancer cells Oncotarget. 2015;6(41):43310-25.

19.	Zhang F, Zhang JG, Qu J, Zhang Q, Prasad C, Wei ZJ. Assessment of anti-cancerous potential of 6-gingerol (Tongling white ginger) and its synergy with drugs on human cervical adenocarcinoma cells. Food and Chemical Toxicology. 2017b;109(Pt 2):910-22.

20.	Chakraborty D, Bishayee K, Ghosh S, Biswas R, Mandal SK, Khuda-Bukhsh AR. [6]-Gingerol induces caspase 3 dependent apoptosis and autophagy in cancer cells: Drug-DNA interaction and expression of certain signal genes in HeLa cells. European Journal of Pharmacology. 2012;694(1-3):20-9.

21.	Zhang F, Thakur K, Hu F, Zhang JG, Wei ZJ. 10-Gingerol, a phytochemical derivative from "Tongling white ginger", inhibits cervical cancer: Insights into the molecular mechanism and inhibitory targets. Journal of Agricultural and Food Chemistry. 2017a;65(10):2089-99.

22.	Pei X, He Z, Yao H, Xiao J, Li L, Gu J, et al. 6-Shogaol from ginger shows anti-tumor effect in cervical carcinoma via Pl3K/Akt/mTOR pathway. European Journal of Nutrition. 2021;60:2781-93.

23.	Liu Q, Peng Y, Qi L, Cheng X, Xu X, Liu L, et al. The cytotoxicity mechanism of 6-shogaol-treated HeLa human cervical cancer cells revealed by label-free shotgun proteomics and bioinformatics analysis. Evidance-Based Complementary and Alternative Medicine. 2012;2012.

24.	Yoysungnoen-Chintana P, Bhattarakosol P, Patumraj S. Antitumor and antiangiogenic activities of curcumin in cervical cancer xenografts in nude mice. Biomed Research International. 2014;2014:817972.

25.	Kapoor V, Aggarwal S, Das SN. 6-Gingerol mediates its anti tumor activities in human oral and cervical cancer cell lines through apoptosis and cell cycle arrest. Phytotherapy Researh. 2016;30(4):588-95.

26.	Carneiro CFD, Drude N, Hülsemann M, Collazo A, Toelch U. Mapping strategies towards improved external validity in preclinical translational research. Expert Opinion on Drug Discovery. 2023;18(11):1273-85.

27.	He Y, Zhu Q, Chen M, Huang Q, Wang W, Li Q, et al. The changing 50% inhibitory concentration (IC 50 ) of cisplatin: a pilot study on the artifacts of the MTT assay and the precise measurement of density-dependent chemoresistance in ovarian cancer. Oncotarget. 2016;7(43).

28.	Haibe-Kains B, El-Hachem N, Birkbak NJ, Jin AC, Beck AH, Aerts HJ, et al. Inconsistency in large pharmacogenomic studies. Nature. 2013;504(7480):389-93.

29.	Wang L, Xi C, Liu R, Ye T, Xiang N, Deng J, et al. Dual targeting of Mcl-1 and Bcl-2 to overcome chemoresistance in cervical and colon cancer. Anti-Cancer Drugs. 2024;35(3):219-26.

30.	Tahmasebi S, Saeed BQ, Temirgalieva E, Yumashev AV, El-Esawi MA, Navashenaq JG, et al. Nanocurcumin improves Treg cell responses in patients with mild and severe SARS-CoV2. Life Sci. 2021;276:119437.

31.	Izadi M, Sadri N, Abdi A, Zadeh MMR, Jalaei D, Ghazimoradi MM, et al. Longevity and anti-aging effects of curcumin supplementation. Geroscience. 2024;46(3):2933-50.

32.	Al-Haideri M, Tondok SB, Safa SH, maleki AH, Rostami S, Jalil AT, et al. CAR-T cell combination therapy: the next revolution in cancer treatment. Cancer Cell International. 2022;22(1):365.

33.	Tahmasebi S, Elahi R, Khosh E, Esmaeilzadeh A. Programmable and multi-targeted CARs: a new breakthrough in cancer CAR-T cell therapy. Clinical and Translational Oncology. 2021;23(6):1003-19.
