# Supplementary material for: Current evidence and future direction on evaluating the anticancer effects of curcumin, gingerols, and shogaols in cervical cancer: A systematic review
Source: PLoS One. 2024 Nov 22;19(11):e0314280. doi: 10.1371/journal.pone.0314280 (PMC11584093; doi:10.1371/journal.pone.0314280)
Supplement: Attachment — Submitted filename: PLOS One-Reviewer comments.docx [file pone.0314280.s005.docx]

Comments to the author:

The current study The paper evaluates the anticancer effects of curcumin, gingerols, and shogaols specifically in cervical cancer and concludes that these compunds have promising anticancer properties. It can be said that the purpose of this study and its content are attractive and interesting. It offers a good perspective for improving the treatment of cervical cancer with natural products. The article is well written and the design of the study and analyzes are done appropriately and correctly. However, to improve the quality of this study, some major points should be addressed and corrected, which are as follows:

Major points:

1. The discussion mentions that most studies included were in vitro, which might not fully represent in vivo conditions. Could you elaborate on how this limitation impacts the generalizability of the findings to clinical settings?

2. The methodological quality of the in vitro studies was assessed as low. How does this low methodological quality affect the reliability of the overall conclusions drawn from the review?

3. There any significant inconsistencies in study designs, such as variations in cell lines, exposure times, or concentrations of bioactive compounds, that could have influenced the results. How were these addressed in the review?

4. Given the challenges in determining an optimal IC50 due to varying cell lines, exposure durations, and assay conditions, how should future research standardize these parameters to enhance comparability?

5. The study may not have fully explored the potential synergistic effects of combining these compounds with each other or with other therapeutic agents.

6. Challenges may exist related to the isolation and purification of these compounds for study purposes. How might these challenges not affect the reproducibility and scalability of research findings?

7. Were there any potential biases in the selection or interpretation of studies that the authors may not have fully addressed? How might these biases impact the conclusions drawn in the review?

8. Given the potential for chemoresistant cervical cancer cells to develop, how might one be sure about these bioactive compounds' effectiveness in overcoming resistance mechanisms?

9. As mentioned in this article, curcumin is a natural nutrient with high potential, which is used as a strong anti-inflammatory substance. Please mention the wide use of curcumin in various diseases (infections, agaeing, cancers, and autoimmunities) in the introduction of a discussion and please consider including the following references in this article (doi:10.1007/s11357-024-01092-5, doi: 10.1016/j.lfs.2021.119437, and doi: 10.1016/j.ejphar.2022.175267 ).

10. The use of emerging and powerful treatments based on immunotherapy, such as tuberculosis therapy, has also improved the survival of patients. In the introduction section, explain about this type of treatment in a few sentences and consider including the following references (10.1186/s12935-022-02778-6, doi: 10.1007/s12094-020-02490-9, and doi: 10.1186/s12935-023-02923-9 ).

11. Another important issue that must be addressed is the presence of inflammatory cytokines in the tumor microenvironment and their confrontation with the PPAR marker due to the existence of common signaling pathways (“Activation of PPARγ negatively influences the production of inflammatory cytokines such as tumor necrosis factor-alpha (TNFα), Interleukin (IL)-6, and IL-1β by macrophages”.). Regarding the contrast with this, explain and consider including the following references in “Cancer Cell Proliferation and Viability” or “Cancer Cell Apoptosis” sections (doi: 10.1007/978-3-030-80962-1_25-1 and doi: 10.1155/2008/961753).

12. The quality and resoultion of all figures are very less, pleasese increase their quality.

13. There are some grammaticlal errors that shoud be corrected throughout the manuscript.
